# Protocol for the *Osteoporosis Choice *trial. A pilot randomized trial of a decision aid in primary care practice

**DOI:** 10.1186/1745-6215-10-113

**Published:** 2009-12-10

**Authors:** Laurie J Pencille, Megan E Campbell, Holly K Van Houten, Nilay D Shah, Rebecca J Mullan, Brian A Swiglo, Maggie Breslin, Rebecca L Kesman, Sidna M Tulledge-Scheitel, Thomas M Jaeger, Ruth E Johnson, Gregory A Bartel, Robert A Wermers, L Joseph Melton, Victor M Montori

**Affiliations:** 1 Knowledge and Encounter Research Unit, Mayo Clinic, Rochester, MN, USA; 2 Division of Biomedical Statistics and Informatics, Department of Health Sciences Research, Mayo Clinic, Rochester, MN, USA; 3 Division of Health Care Policy and Research, Department of Health Sciences Research, Mayo Clinic, Rochester, MN, USA; 4 Endocrinology, United Medical Specialties, Allina Medical Clinic, St. Paul, MN, USA; 5 SPARC Design Studio, Center for Innovation, Mayo Clinic, Rochester, MN, USA; 6 Division of Primary Care Internal Medicine, Department of Internal Medicine, Mayo Clinic, Rochester, MN, USA; 7 Division of Preventive, Occupational, and Aerospace Medicine, Department of Medicine, Mayo Clinic, Rochester, MN, USA; 8 Department of Family Medicine, Mayo Clinic, Rochester, MN, USA; 9 Division of Endocrinology, Diabetes, Metabolism, and Nutrition, Department of Internal Medicine, Mayo Clinic, Rochester, MN, USA; 10 Division of Epidemiology, Department of Health Sciences Research, Mayo Clinic, Rochester, MN, USA

## Abstract

**Background:**

Bisphosphonates can reduce fracture risk in patients with osteoporosis, but many at-risk patients do not start or adhere to these medications. The aims of this study are to: (1) preliminarily evaluate the effect of an individualized 10-year osteoporotic fracture risk calculator and decision aid (*OSTEOPOROSIS CHOICE*) for postmenopausal women at risk for osteoporotic fractures; and (2) assess the feasibility and validity (i.e., absence of contamination) of patient-level randomization (vs. cluster randomization) in pilot trials of decision aid efficacy.

**Methods/Design:**

This is a protocol for a parallel, 2-arm, randomized trial to compare an intervention group receiving *OSTEOPOROSIS CHOICE *to a control group receiving usual primary care. Postmenopausal women with bone mineral density T-scores of <-1.0, not receiving bisphosphonate therapy, and receiving care at participating primary care practices in and around Rochester, Minnesota, USA will be eligible to participate in the trial. We will measure the effect of OSTEOPOROSIS CHOICE on five outcomes: (a) patient knowledge regarding osteoporosis risk factors and treatment; (b) quality of the decision-making process for both the patient and clinician; (c) patient and clinician acceptability and satisfaction with the decision aid; (d) rate of bisphosphonate use and adherence, and (e) trial processes (e.g., ability to recruit participants, collect patient outcomes). To capture these outcomes, we will use patient and clinician surveys following each visit and video recordings of the clinical encounters. These video recordings will also allow us to determine the extent to which clinicians previously exposed to the decision aid were able to recreate elements of the decision aid with control patients (i.e., contamination). Pharmacy prescription profiles and follow-up phone interviews will assess medication start and adherence at 6 months.

**Discussion:**

This pilot trial will provide evidence of feasibility, validity of patient randomization, and preliminary efficacy of a novel approach -- decision aids -- to improving medication adherence for postmenopausal women at risk of osteoporotic fractures. The results will inform the design of a larger trial that could provide more precise estimates of the efficacy of the decision aid.

**Trial registration:**

Clinical Trials.gov Identifier: NCT00578981

## Background

The societal burden associated with osteoporotic fractures is already great[1], but is expected to increase further with aging of the population [2]. Randomized trials have conclusively shown that use of bisphosphonate therapy in postmenopausal women can reduce their risk of osteoporotic fractures. Postmarketing studies have established a favorable balance of benefit over harms [3]. Limited start and adherence to bisphosphonates in at-risk patients, however, reduces this beneficial impact [4-7]. When bisphosphonates are prescribed, about 50% of patients discontinue therapy within one year of its prescription [8,9]. Given current estimates of the prevelance of at-risk people -- the National Osteoporosis Foundation estimates that 44 million people in the United States age 50 years or over have either osteoporosis or osteopenia [10] -- the potential morbidity, mortality and expense associated with bisphosphonate nonadherence is significant.

Poor adherence appears related, in part, to undisclosed and unexplored patient values and preferences [11,12], as well as gaps in patient understanding of the efficacy and safety of pharmacologic treatment [13]. One way to facilitate patient understanding and to elicit patient preferences is to use decision aids in consultations. Decision aids are tools that help patients participate in choosing among management options by providing them with information about the relevant features of the available choices. A systematic review of 55 randomized trials of decision aids vs. usual care, pamphlets, or education revealed that, overall, decision aids are effective at improving knowledge acquisition and reducing so-called decisional conflict (i.e., uncertainty about the choice, ignorance about the pros and cons of each option, pressure to make a particular choice, and effectiveness of the decision) [14]; their effect on medication adherence remains largely unknown.

To our knowledge, our group has produced the only randomized trial evidence supporting improvements in adherence to preventive medications, using a decision aid about statins in patients with type 2 diabetes [15]. In this case, clinicians and patients found the tool, *Statin Choice*, acceptable; and, compared with no intervention, patients receiving the decision aid had more knowledge about and more accurate expectations of potential benefits and harms of statin use. They also reported significant reductions in decisional conflict and showed a 3-fold increase in the odds of self-reported adherence to statins at 3 months.

The timing for *OSTEOPOROSIS CHOICE *has coincided with a paradigm shift in the management of osteoporosis [10]. The prior standard approach relied most heavily on bone mineral density (BMD) results as measured with dual-energy X-ray absorptiometry (DXA) to inform practice guidelines and treatment decisions [16,17]. While BMD T-scores can identify high risk patients, they are both insensitive and nonspecific [18] since they do not address other pertinent factors related to risk of fracture [19]. Moreover, the actual probability of experiencing an osteoporotic fracture with any given T-score is not immediately evident or available [20]. With the arrival of FRAX, the World Health Organization's calculator of 10-year osteoporotic fracture risk [21], treatment recommendations can now be commensurate with the probability of a hip fracture or any major osteoporotic fracture (hip, spine, distal forearm, humerus). FRAX risk estimates are based on population-based cohort studies. These studies generated models that incorporate a patient's BMD at the femoral neck, when available, and clinical risk factors. These factors include age, sex, ethnicity, body mass index, personal and parental history of prior osteoporotic fracture, long term use of oral glucocorticoids, rheumatoid arthritis or other secondary causes of osteoporosis (e.g. type 1 diabetes, premature menopause, chronic malabsorption, hyperthyroidism, hypogonadism), and current alcohol and tobacco use [19]. Current osteoporosis practice guidelines incorporate the use of FRAX [10], but optimal use of this tool is still evolving [20].

At the onset of this project, the best available decision aid for osteoporosis treatment was 35 pages long [22], did not incorporate absolute fracture risk estimates, and its efficacy remained unexplored. In contrast, *OSTEOPOROSIS CHOICE*:

• is a one page, paper-based decision aid;

• incorporates a specific patient's 10-year risk of an osteoporotic fracture estimated using the FRAX online calculator http://www.shef.ac.uk/FRAX/; and

• is designed for use between patients and their clinicians.

The aim of this study is to evaluate the effect of an individualized fracture risk calculator and decision aid for postmenopausal women at risk of osteoporotic fractures, *OSTEOPOROSIS CHOICE*, on knowledge transfer, the quality of the decision making process, and on patient adherence to medications. Given our choice of study design, i.e., randomization at the patient level, we also will seek to evaluate the potential for contamination and poor fidelity in the use of the decision aid in this setting. Here we present the protocol we are using to conduct this trial.

## Methods

### Design

To evaluate the decision aid, we propose to conduct a patient-level multicenter randomized trial. The Mayo Clinic Institutional Review Board approved all study procedures.

### Setting

Patients and primary care clinicians for this trial are to be recruited at 10 practice sites affiliated with the Mayo Clinic, all located in Southeastern Minnesota within a 60-mile radius of Rochester, Minnesota, USA.

### Participants

#### Inclusion criteria

Eligible clinicians include physicians, physician assistants and nurse practitioners at participating sites. Eligible patients include postmenopausal women, age 50 and over who have BMD levels consistent with a diagnosis of low bone mass (osteopenia) or osteoporosis, who are not taking bisphosphonates or other prescription osteoporosis medications to treat low bone mass (other than vitamin D and calcium), whom their clinicians find eligible for bisphosphonate therapy and have a follow-up appointment with their clinician, and who are available for a telephone follow-up 6 months after randomization.

#### Exclusion criteria

*The trial will exclude *women who cannot read English or have, in their clinicians' judgment, major learning barriers such as visual or hearing impairment or dementia that would compromise their ability to give written informed consent (or use the decision aid).

#### Participant recruitment

We will identify eligible women from DXA lists and from participating clinicians' appointment calendars. If there were no pre-scheduled follow-up appointments after the BMD evaluation, we will request participating clinicians schedule such an appointment to discuss abnormal BMD results with the patient during an office visit, not via a telephone conversation. The study coordinator will inform patients about the trial, confirm their eligibility, and obtain written informed consent in a private room at the clinic site. Patients will be offered 15 dollars in compensation for time spent completing study procedures.

### Interventions

#### Decision aid

The *OSTEOPOROSIS CHOICE *decision aid tool was developed through an iterative process and with extensive field-testing involving patients, clinicians, designers, and researchers [23,24]. This one-page decision aid provides the patient's individualized 10-year risk estimate of having a major osteoporotic fracture (clinical spine, forearm, hip or shoulder fracture). The risk is calculated using data from the patient's medical record (completed by direct query to patient or clinician as needed) and entering it into the online FRAX tool as implemented during the course of the study.

We developed three decision aids that describe the pros and cons of treatment, one for each of three categories of estimated 10-year risks of fracture: <10%, 10-30%, and >30%. At the time we designed the decision aids, there were no guidelines to orient therapeutic decisions based on fracture risk. Thus, the design team, in conjunction with patients and clinicians, arbitrarily chose these thresholds. The decision aid also shows the patient-specific absolute reduction of fracture risk with alendronate, assuming a treatment-related reduction in overall osteoporotic fracture risk of 40% [25], and the potential downsides of taking bisphosphonates.

**Figure 1:**
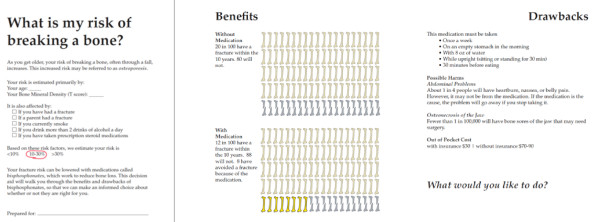
**Osteoporosis Choice decision aid for a patient with a 10-year fracture risk of 20%**.

To estimate the gastrointestinal side effects of oral bisphosphonates we reviewed the FDA-approved package insert, reports of pivotal clinical trials, and observational studies [3]. Evidence of the association between bisphosphonate use and osteonecrosis of the jaw was also evolving while we were designing the tool [26]. The estimate presented was derived from available reviews of weak evidence and expert judgment from specialist clinicians on our research team [27]. Finally, the decision aid also prompted further discussion of these issues with the question "What would you like to do? (Figure 1).

In the intervention arm, a study coordinator will review with the clinician the patient's tailored *OSTEOPOROSIS CHOICE *decision aid tool immediately prior to the clinician's visit with that patient. The clinician and the patient will then review the decision aid together in the context of their clinical encounter. This procedure, which is similar to one used in our prior decision aid trial in primary care [28], has the advantages of being time-sensitive and incorporates as-needed just-in-time training in the use of the tool. We have found that clinicians delivering decision aids during the visit yield somewhat superior results (e.g. significantly improved knowledge transfer) to researchers delivering the tool prior to the visit [29].

#### Usual care

In the usual care arm, the clinician will discuss the patient's BMD test results and options for treatment in that clinician's usual fashion, i.e., with no other research-related intervention. The patient will receive the National Osteoporosis Foundation booklet, "Boning Up On Osteoporosis: A Guide To Prevention and Treatment."

### Randomization

A computer-generated allocation sequence will randomize patients 1:1 in a concealed fashion to control (usual care + booklet) or intervention (*OSTEOPOROSIS CHOICE *decision aid). The study coordinator will complete the following steps:

• assess the eligibility of the patient, obtain the patient's written informed consent and enroll the patient;

• access a secure study website, input patient identifiers to document patient eligibility;

• obtain from the website interface the arm of the study to which the patient is allocated;

• when the website is not available, the coordinator will contact the study statistician and a similar procedure will be used to provide concealed random allocation.

After randomization, only data analysts will remain blind to allocation. If a patient were suspected of having incorrectly enrolled, e.g., the patient is found to be ineligible after enrollment and randomization, the study coordinator will present the case to the study principal investigator (V.M.M.) for consideration. Following existing recommendations [30], the principal investigator will decide on exclusion while unaware of any consequences of enrollment, including the patient's allocation or outcomes.

The following issues were considered in our choice[15] to randomize clinics, clinicians, or patients:

• *Randomize by clinics *- This approach would enable us to train everyone in the intervention (and control) clinics to use (or not) the decision aid. Disadvantages included the small number of available clinics, difficulty in balancing the number of participating clinicians and patients across sites, and limited ability to avoid selection bias or implement allocation concealment.

• *Randomize by clinicians *- This approach would enable clinicians to develop skills for using the decision aid, and resolve any confusion related to when each clinician should use or not use the decision aid. Unfortunately, in our judgment, these advantages did not outweigh the inability to implement allocation concealment, the residual potential for selection bias, and the potential for a large imbalance of numbers of patients per clinician that could emerge [15]. Because of the relatively small number of participating clinicians, clinician charisma or communication skills could confound the results of the effect of the decision aid. Finally, there could be a potential risk of recruitment and randomization of clinicians with few or null eligible or enrolled patients during the trial.

• *Randomize by patients *- This approach would enable allocation concealment and avoid selection bias. However, it opens the study to the potential for contamination at the clinician level; it is difficult logistically to determine when a clinician will use the decision aid or not (and therefore expedite recruitment in a busy practice setting); and it slows the learning curve that may exist when clinicians adapt to using the decision aid in practice.

Because the design was simple and we placed a high value in avoiding bias, we implemented this third approach. The main disadvantage of patient randomization is clinician contamination. Contamination in this case refers to a clinician who, having used the decision aid with a prior patient, is able to recreate elements of the decision aid with a subsequent patient allocated to receive usual care. We use the term 'recreate' given that clinicians will not have access to the decision aid when seeing patients not allocated to the intervention arm. We recognize that clinicians could produce these elements by drawing from their own knowledge base, i.e., not true contamination, so to this extent this assessment may overestimate the rate of contamination.

Since we did not find a published approach to determine the extent to which this design could introduce contamination, we developed a "contamination checklist" with which to judge how clinicians will present information about osteoporosis risk and treatment effect of bisphosphonates in video recordings of the clinical encounters (see below). The tool checks for clinicians' use of 12 key elements of the decision aid (Additional File 1) when seeing a control patient. A score of 12 in a control visit with a clinician who has previously used the decision aid would indicate maximum contamination (note that a score of 12 in an intervention patient would indicate maximum fidelity in delivering the decision aid). Given the small sample size and number of clinicians likely to enroll more than one patient, only a few instances of potential contamination (i.e., control patients visit with a clinician who has had a decision aid visit previously) are likely to take place. Therefore, our analyses will be limited to descriptions of the distribution of overall scores and the frequency of each item.

### Outcomes and data collection

In this pilot trial, we will note the success and viability of the trial processes, including the proportion of eligible patients and clinicians we are able to recruit and our ability to collect and analyze all outcomes in all randomized patients, i.e., to adhere to the intention to treat principle.

To assess efficacy, we will measure the effect of *OSTEOPOROSIS CHOICE *on the following outcomes: (a) patient knowledge about osteoporosis risk factors and treatment; (b) quality of the decision-making process for patient and clinician; (c) patient and clinician acceptability and satisfaction with the decision aid; and (d) rate of bisphosphonate use and adherence in at-risk women. We will survey patients and clinicians after each visit (the complete set of questions is in the **appendix) **and video record the clinical encounters to capture these outcomes. Pharmacy prescription profiles and follow-up telephone interviews will assess medication start and adherence at 6 months.

The patient questionnaire includes:

• Items about education level, annual income and health status for descriptive purposes.

• 15 items to assess patient knowledge, 13 of which refer to osteoporosis in general and the benefits and downsides of taking a bisphosphonate (9 questions about information on the decision aid, 4 questions not informed by content on the decision aid), as well as two questions about patient's personalized fracture risk and estimated risk reduction with bisphosphonates. These items were fashioned after similar items showed construct validity and responsiveness to the intervention in previous decision aid trials [14,15,28].

• 5 items using a 7-point Likert-type scale to assess the patient's perceptions of the amount, clarity and helpfulness of information, their desire to receive information about other medical treatment choices in the same way this information was delivered, and whether they would recommend the way they received information from their clinician during this visit to other patients. We have used these same items in our previous trials [15,28].

• 10 items from the Trust in Physician scale [31] and

• 16 items from the Decisional Conflict Scale [32] -- the most commonly used outcome measure in clinical trials of decision aids, which assesses the extent to which the decision was informed, consistent with values, free of pressure, and effective.

• Additional questions (see appendix) inquire about the treatment decision made during the visit and patient satisfaction with the degree of participation in the decision-making process.

The clinicians' survey, to be completed after each study visit, includes questions regarding which decision the patient made, the clinician's confidence in the patient's understanding of the information offered, and the clinician's prediction of patient action (start and adherence to bisphophonates at 6 months).

To evaluate the quality of decision-making, we will use the OPTION scale [33] on the video recordings. This scale allows an observer to quantify the extent to which clinicians are able to involve patients in the decision-making process. We have used the OPTION scale on video-recorded encounters with adequate reliability in previous studies [28,34]. Video recordings also provide information about encounter duration (using the video time stamps), fidelity of use of the tool, and difficulties and successes experienced while using the decision aid, and are the source of data for the contamination checklist described above.

We will use two methods to assess patient adherence to bisphophonates at six months. We will contact patients by telephone and measure patient self-reported adherence using the Haynes' single item adherence question ("Have you missed any of your pills in the last week?") a measure associated with a 96% specificity and 75% accuracy vs. a pill count standard [35,36]. We will also obtain patients' pharmacy records in order to objectively determine medications filled since the study visit. Using this information, we will consider a gap (time without medication) of longer than 90 days as bisphosphonate discontinuation. We will assess adherence to bisphosphonates by assessing the proportion of days covered during the 180 days after the visit, taking into account potential overlap in supply that may result when a refill comes before the estimated completion of the previous fill. We will define persistence as the number of days from the first prescription fill to the last fill in the 180 days after the visit. In this case, we will take into account overlapping supply and the number of days supplied at the last fill, truncating at 180 days after the visit [37,38]. In addition to these continous measures, we will assess and compare the proportion of patients who exhibited ≥ 80% adherence to bisphosphonates in both groups.

### Statistical considerations

We have funding and plan to enroll and randomize 100 patients. While this number of patients is insufficient to reach a definitive answer about the efficacy of the decision aid and its effect on medication adherence, it is sufficient to ascertain the following differences:

• for knowledge gains, assuming that patients in the control group will be able, on average, to answer 4 of the 9 specific knowledge questions correctly, we will have 92% power to detect a ≥42.5% increase in mean knowledge (to a mean of 5.6 questions answered correctly) in the intervention group, assuming equal variances between the two arms and alpha = 0.05;

• for adherence, estimating that the 6 month adherence rate is 65% (null hypothesis), we will be powered (80%) to detect a difference (alpha = 0.05) in adherence rates of 35% (88% alternative hypothesis). To our knowledge, there is no policy- or patient-important minimum difference in adherence rates identified in the literature to otherwise guide the choice of a threshold.

The distribution of study variables will be described (using frequencies for categorical variables and measures of central tendency for continous variables) and compared using relative (relative risk) or absolute (mean differences) measures of association and their 95% confidence intervals. Because we expect a very low patient-to-clinician ratio, we do not anticipate having to use generalized estimating equations to adjust for clustering by clinician. To test hypotheses of association, we will use Wilcoxon rank-sum tests to compare medians, and Chi-square or Fisher's Exact tests to compare frequencies. All analyses are based on two-sided tests at significance level 0.05. Consistent with the intention to treat principle [39], all efforts will be placed on avoiding missing data and all patients will be analyzed in the arm to which they were randomized; participants with missing data will not be part of the analyses requiring the missing data. All analyses will be conducted using SAS (SAS Institute, Cary, NC).

## Discussion

We have presented the design of a trial to evaluate the effect of a decision aid for bisphosphonate treatment in postmenopausal women receiving primary care (*OSTEOPOROSIS CHOICE*). While this is a feasibility trial for a new decision aid, we also seek to determine if the decision aid can transfer knowledge, and if so, can it improve patient involvement in making decisions about their care, and whether such an improvement will have a discernible effect on adherence. We also discuss the pros and cons of patient randomization vs. cluster randomization, and present a method using video recordings of visits to assess contamination, a key potential outcome of our decision to randomize by patients, rather than clinics or clinicians.

Pursuing adherence as an outcome of decision support technologies is pertinent for clinical policymaking, but it is challenging from a clinical trials design standpoint, especially when few patients take up the medication of interest after the consultation. In this case, the power of a trial to measure differences in this outcome depends not only on the efficacy of the intervention, but also on the proportion of patients at risk of nonadherence. The latter depends on the fraction of patients who leave the consultation with a prescription, a number that could be substantially smaller than the number randomized. Most cohort studies of medication use cannot ascertain the patients who had a discussion about medication use and left the consultation without a prescription. Consequently, the proportion of patients who will begin a new medication is often unknown prior to the study. This pilot study will offer an estimate of the proportion of patients likely to take on bisphosphonates after the study visit, this estimate can inform the size of larger trials of this intervention seeking to evaluate the impact of decision aids on adherence to medication.

An important reason to run feasibility trials of decision aids designed for use in clinical practice is that these tools could directly impact the content and duration of the consultation in favorable or unfavorable ways. When clinicians at multiple sites with different interests and priorities see eligible patients, they may not perceive the need to use the decision aid for this particular decision, may not find it appropriate for use in some patients, and may not use the decision aid appropriately [40]. Thus, an evaluation of the feasibility of the decision aid aided by video recording of as many visits as possible provides a rich database to ascertain these challenges. Results may lead to process modifications in trial design, revised criteria for selection of clinicians and patients, or changes in the decision aid itself, prior to a larger efficacy study. These insights will also affect efforts to implement effective decision aids in practice [41,42].

In conclusion, the *OSTEOPOROSIS CHOICE *trial is designed to provide initial empirical evidence of feasibility and efficacy of a novel approach -- decision aids -- to improve adherence to medication for patients with osteoporosis. We will also seek to assess the feasibility and effect on validity of patient-level randomization as an alternative to cluster randomization at the clinician and clinic level in preliminary studies of decision aid efficacy. The results will inform the design of a larger trial that could provide more precise estimates of the efficacy of the decision aid.

## List of abbreviations

BMD: (bone mineral density); DXA: (dual-energy X-ray absorptiometry); FRAX: (fracture risk assessment tool); WHO: (World Health Organization).

## Competing interests

The authors declare that they have no competing interests.

## Authors' contributions

LP contributed to the design of the study, designed study procedures, and wrote the first draft of this manuscript. MC and HV contributed to the statistical analysis of the study, wrote the statistical section of this manuscript and provided critical revisions to other sections of this manuscript. NS contributed to the design of procedures to ascertain medication adherence and to conduct the statistical analysis of these data, as well as provided critical revisions to this manuscript. RM contributed to trial procedures and provided critical input and edits to the manuscript. BS conceived the study, and participated in its design, and made critical revisions to the manuscript. MB designed the decision aid Osteoporosis Choice, and provided input into the protocol and critical revisions to this manuscript. RK, STS, TJ, RJ, and GB contributed to the refinement of the enrollment and trial procedures and provided critical input and edits to the manuscript. RW and LJM contributed to the design of the intervention and the protocol, and made critical revision of the manuscript. VM made substantial contributions to conception and design of the study, to the study protocol, and made critical revisions to the manuscript. All authors approved the final version of this manuscript.

## Supplementary Material

Additional file 1 **Contamination checklist**. The file contains the contamination checklist to be applied to video recorded encounters. Click here for file
