# Supplementary material for: Protocol for the Osteoporosis Choice trial. A pilot randomized trial of a decision aid in primary care practice
Source: Trials. 2009 Dec 10;10:113. doi: 10.1186/1745-6215-10-113 (PMC2796658; doi:10.1186/1745-6215-10-113)
Supplement: Additional file 1 — Contamination checklist. The file contains the contamination checklist to be applied to video recorded encounters. [file 1745-6215-10-113-S1.DOC]

**Contamination checklist -** Study personnel working in duplicate will use this checklist while reviewing video recordings of the clinical encounters in the trial to determine the extent to which clinicians were able to complete the actions noted with control patients. The extent to which clinicians are able to recreate the content of the decision aid with control patients after having the opportunity to use the decision aid would indicate contamination. Incidentally, the checklist will also indicate the extent to which clinicians are able deliver the decision aid with adequate fidelity.

| **Baseline Risk** |
| --- |
| 1. Did the clinician describe the risk of breaking a bone numerically? |
| 2. Did the clinician describe the risk as a natural frequency (*e.g.* *“of 100 people like you, 6 will…”*) ? |
| 3. Did the clinician describe the time horizon for risk of breaking a bone ? |
| 4. Did the clinician describe the risk graphically? |
| **Risk Reduction (from bisphosphonates)** |
| 5. Did the clinician describe the risk reduction numerically? |
| 6. Did the clinician describe risk reduction as a natural frequency **(**e.g. *“if 100 people take the medication, 10 will . . . ”*)? |
| 7. Did the clinician describe the time horizon for the risk reduction? |
| 8. Did the clinician describe the risk reduction graphically? |
| **Side Effects** |
| 9. Did the clinician describe the frequency of gastrointestinal side effects associated with bisphosphonates? |
| 10. Did the clinician describe the frequency of osteonecrosis of the jaw associated with bisphosphonates? |
| **Risk Factors** |
| 11. Did the clinician identify any of the following factors as a risk factor for the patient’s likelihood of breaking a bone: age, prior fracture, parental fracture, alcohol consumption, smoking, corticosteroid use. |
| **Cost** |
| 12. Did the clinician describe the cost of the medication? |
